# Why Physicians Delay in the Diagnosis and Treatment of Valproic Acid Toxicity

**DOI:** 10.7759/cureus.100436

**Published:** 2025-12-30

**Authors:** Muhammad Shahid, Amber Asghar, Diane L Levine

**Affiliations:** 1 Medicine, Wexham Park Hospital, Slough, GBR; 2 Internal Medicine, Detroit Medical Center, Wayne State University, Detroit, USA

**Keywords:** alcohol, diagnostic delay, lactulose, levocarnitine, valproic acid toxicity

## Abstract

Objective: This study aims to review valproic acid (VA) toxicity and treatment at an urban safety-net hospital to determine the timeliness and appropriateness of treatment and outcomes.

Methods: We gathered data on 159 patients with the diagnosis of VA toxicity who were admitted to the Detroit Medical Center (USA) from January 2005 to 2015. Patients with known liver disease due to alcohol intake and viral infections were excluded. We extracted the following parameters and compared them: demographic variables, time at presentation, time elapsed between presentation and diagnosis, time elapsed between diagnosis and treatment, time elapsed between treatment and discharge, the total hospital length of stay (LOS), diagnosis, symptoms, treatments given, concomitant medications, and laboratory values. The analysis of all variables was descriptive, and simple correlations were done between dependent and independent variables.

Result: A total of 159 patients with VA toxicity were identified. The majority of patients (53% male, 47% female) were African Americans (75%), with the remainder Caucasian (22%) and others (3%). According to our data, one-third (33%) of the total cases comprised patients who had multiple disorders (schizophrenia, depression, bipolar, seizure), 36 (22.6%) had schizophrenia, 34 (21%) had bipolar disorder, 23 (14%) had seizure disorder, 11 (7%) had major depression, and two (1%) were without any previous diagnosis. Data showed that 72 patients (45.3%) were found with altered mental status, 49 (31%) with GI and other CNS symptoms, and 38 (24%) without any symptoms. There were 58 (37%) patients who committed a suicidal attempt, but more than half were asymptomatic (59%) on initial presentation. Disposition included discharge from ED in 22 (15%), admission to psychiatry in 11 (7%), floors in 108 (68%), and ICU in 18 (11%). The mean time from presentation to diagnosis was three hours, with a range of 0.1-10 hrs. Supportive care alone was given to 62 (39%), charcoal to 35 (22%), and pharmacological treatment to 62 (39%). Of those receiving pharmacologic treatment, 47 received only levocarnitine; 15 received lactulose followed by levocarnitine. The average ammonia level at presentation among those who received pharmacologic treatment was 89 ± 63 mcg/dL. Those treated with lactulose (15/62) had an initial ammonia level of 97 ± 62 mcg/dL, which fell to 72 ± 58 mcg/dL at 20 hrs. Once levocarnitine was added, ammonia decreased to 56 ± 25 mcg/dL at 25 hrs. Those who were treated only with levocarnitine had an initial ammonia level of 87 ± 63 mcg/dL, which fell to 64 ± 48 mcg/dL at 14 hrs. Of 159 patients, 44 (28%) had known alcohol abuse. In those who required pharmacologic treatment, the time from diagnosis to appropriate treatment with levocarnitine was 7.8 hrs in alcoholics and 4.3 hrs in patients who were not alcoholics. No patient with elevated alcohol on admission was diagnosed with hepatic encephalopathy on discharge. The average hospital LOS for those who received pharmacologic treatment was 92.4 hrs. Patients who received lactulose had a LOS of 98.9 hrs. The total LOS for those who did not receive lactulose was 90.3 hrs.

Conclusion: Early diagnosis of VA toxicity is necessary for timely, effective, high-quality care. Patients who initially received lactulose for hyperammonemia had slower recovery. Recognition of VA toxicity and prompt initiation of levocarnitine are associated with faster recovery and shorter LOS.

## Introduction

Valproic acid (VA) was initially approved for the treatment of seizures. With approval for mania and migraines, there has been an increase in use and toxicity. VA toxicity is associated with altered mental status, gastrointestinal (GI) symptoms, and hyperammonemia. According to the 2004 and 2008 annual reports of the American Association of Poison Control Centers (AAPCC), 9,000 and 8,462 acute exposures involving VA were reported, respectively [1-3]. Previously, it was reported that identified young age (<2 years), developmental delay, coincidental metabolic disorder, and neurologic diseases concomitant with treatment with other AEDs (antiepileptic drugs) such as phenytoin, carbamazepine, or phenobarbitone are risk factors for toxicity [4-8]. Factors that contribute to a delay in diagnosis and timely treatment include concomitant treatments, the vagueness of symptoms at presentation, hepatic diseases from other causes, normal VA concentration and ammonia levels at presentation, alcohol toxicity, and psychiatric and neurological disorders. Hyperammonemia and hepatotoxicity may be mediated by carnitine and acetyl-CoA deficiency, and early intervention with levocarnitine is associated with the greatest survival, although large randomized controlled trials of levocarnitine treatment in encephalopathy are lacking. Levocarnitine has been generally safe and effective in retrospective trials and case reports [9-12].

We aimed to investigate the delay in diagnosing patients with VA toxicity and the effects of starting the proper treatment in a timely manner. Furthermore, our goal is to assess the efficacy of lactulose in reducing ammonia levels in VA toxicity. Ultimately, our work on VA toxicity aims to ensure that patients receive appropriate treatment without delay.

The objective of this study will be to find out the factors that cause delay in the treatment of VA toxicity. The secondary objective is that lactulose is not an effective treatment for VA toxicity regardless of an increased ammonia level.

## Materials and methods

It is a retrospective observational study. We gathered data of 159 patients with the diagnosis of VA toxicity who were admitted to the Detroit Medical Center (USA) during the period of January 2005 to 2015. The study was conducted with the approval of the Wayne State University Institutional Review Board (IRB). Patients were included who were diagnosed with VA toxicity and had a primary diagnosis of mania, schizophrenia, bipolar disorder, depression, or seizures. Patients with known liver disease due to alcohol intake and viral infections were excluded.

We extracted the following parameters and compared them: demographic variables, time at presentation, time elapsed between presentation and diagnosis, time elapsed between diagnosis and treatment, time elapsed between treatment and discharge, the total length of hospital stay, diagnosis, symptoms, treatments given, concomitant medications, and laboratory values. The analysis of all variables was descriptive, and a simple correlation was done between the dependent and independent variables.

## Results

**Figure 1:**
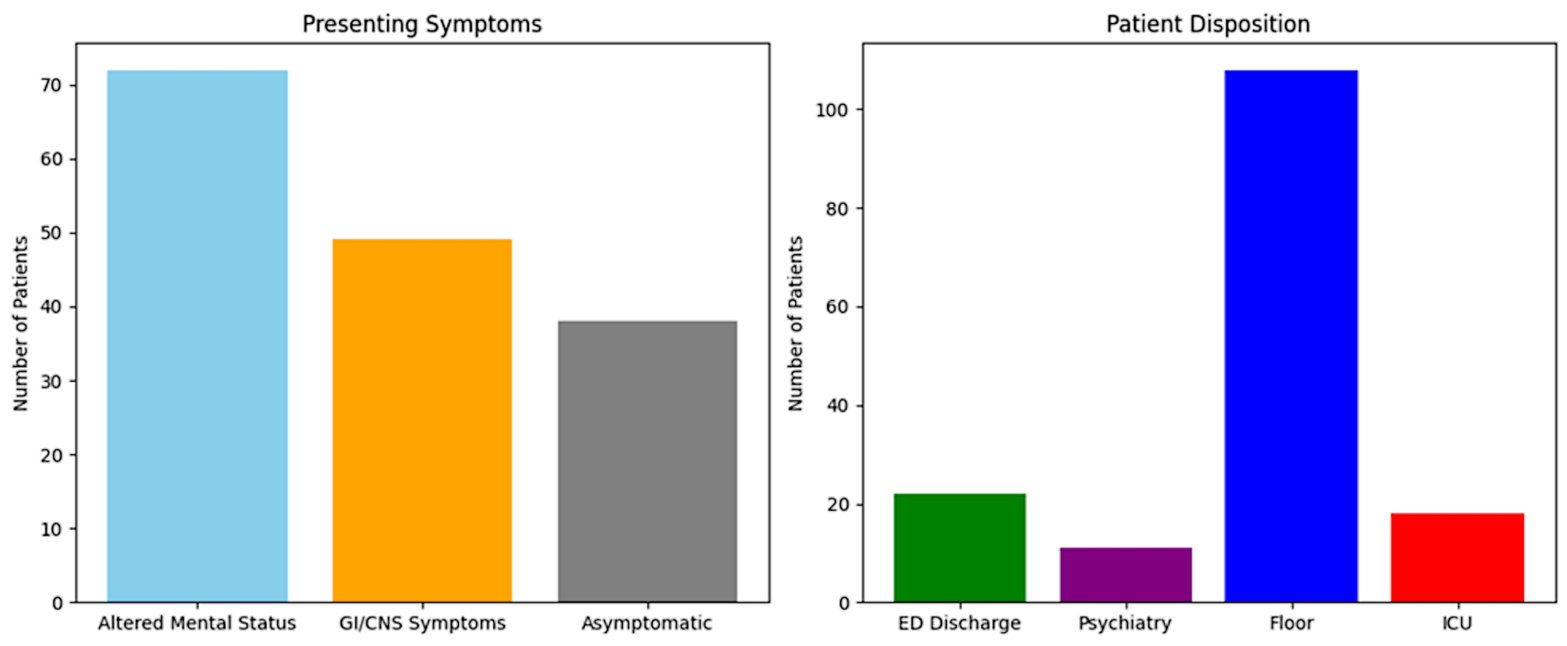
Presenting symptoms and patient disposition The left panel represents presenting symptoms, with altered mental status being the most common (45.3%). The right panel shows patient disposition, with the majority admitted to medical floors (68%). This visualization emphasizes the need for rapid assessment and triage in valproic acid toxicity. GI: gastrointestinal; CNS: central nervous system; ED: emergency department; ICU: intensive care unit

**Figure 2:**
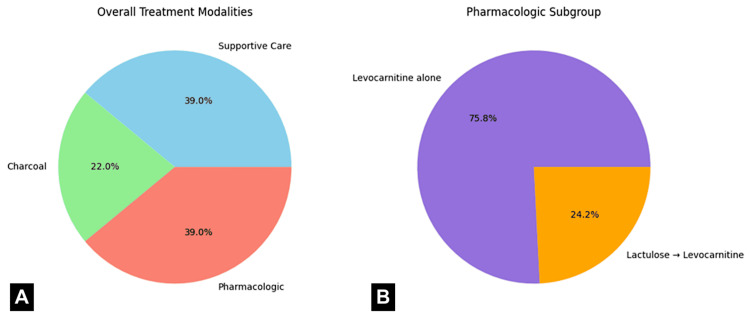
Treatment modalities in valproic acid toxicity (A) Overall treatment modalities: supportive care (39%), activated charcoal (22%), and pharmacologic therapy (39%). (B) Pharmacologic subgroup: levocarnitine alone (47 patients) and lactulose followed by levocarnitine (15 patients). Levocarnitine was the primary effective treatment for hyperammonemia.

A total of 159 patients with VA toxicity were identified. The majority of patients (53% male, 47% female) were African Americans (75%), with the remainder Caucasian (22%) and other (3%). According to our data, one-third (33%) of the total cases comprised patients who had multiple disorders (schizophrenia, depression, bipolar, seizure), 36 (22.6%) had schizophrenia, 34 (21%) had bipolar disorder, 23 (14%) had seizure disorder, 11 (7%) had major depression, and two (1%) were without any previous diagnosis. Data showed that 72 patients (45.3%) were found with altered mental status, 49 (31%) with GI and other CNS symptoms, and 38 (24%) without any symptoms. There were 58 (37%) patients who committed a suicidal attempt, but more than half were asymptomatic (59%) on initial presentation. Disposition included discharge from ED in 22 (15%), admission to psychiatry in 11 (7%), floors in 108 (68%), and ICU in 18 (11%) (Figure 1). The mean time from presentation to diagnosis was three hours (hrs), with a range of 0.1-10 hours (Table 1). Supportive care alone was given to 62 (39%), charcoal to 35 (22%), and pharmacological treatment to 62 (39%) (Figure 2). Of those receiving pharmacologic treatment, 47 received only levocarnitine, and 15 received lactulose followed by levocarnitine.

**Table 1:** Treatment modalities and time to diagnosis Diagnosis was typically established within a few hours of presentation. Treatment strategies varied considerably, with nearly equal proportions receiving supportive care alone or pharmacologic intervention. Levocarnitine was the most frequently used pharmacologic therapy, although lactulose was initially administered in a subset of patients.

Variable	Value
Mean time to diagnosis	3 hours (range 0.1–10)
Supportive care only	62 (39%)
Activated charcoal	35 (22%)
Pharmacologic treatment	62 (39%)
Levocarnitine alone	47
Lactulose → Levocarnitine	15

The average ammonia level at presentation among those who received pharmacologic treatment was 89 ± 63 mcg/dL. Those treated with lactulose (15/62) had an initial ammonia level of 97 ± 62 mcg/dL, which fell to 72 ± 58 mcg/dL at 20 hrs. Once levocarnitine was added, ammonia decreased to 56 ± 25 mcg/dL at 25 hrs. Those who were treated only with levocarnitine had an initial ammonia level of 87 ± 63 mcg/dL, which fell to 64 ± 48 mcg/dL at 14 hrs (Table 2).

**Table 2:** Comparison between ammonia levels before and after treatment n = 62, the number of patients who received pharmacological treatment. Patients treated with levocarnitine alone demonstrated a faster reduction in ammonia levels compared to those initially treated with lactulose. Although lactulose led to a modest reduction in ammonia, a substantial improvement occurred only after levocarnitine initiation, supporting its primary role in treatment.

Treatment Group	Initial Ammonia (mcg/dL)	Follow-Up Ammonia	Time to Reduction
Levocarnitine alone (n=47)	87 ± 63	64 ± 48	14 hours
Lactulose → Levocarnitine (n=15)	97 ± 62	72 ± 58	20 hours
After levocarnitine added	-	56 ± 25	25 hours

Of 159 patients, 44 (28%) had known alcohol abuse (Table 3). In those who required pharmacologic treatment, the time from diagnosis to appropriate treatment with levocarnitine was 7.8 hrs in alcoholics and 4.3 hrs in patients who were not alcoholics. No patient with elevated alcohol on admission was diagnosed with hepatic encephalopathy on discharge. The average hospital length of stay (LOS) for those who received pharmacologic treatment (levocarnitine) was 92.4 hrs. Patients who received lactulose had a LOS of 98.9 hrs.

**Table 3:** Alcohol use, treatment delay, and length of stay Alcohol abuse was associated with delayed initiation of levocarnitine therapy. Despite elevated alcohol levels at presentation, no patient developed hepatic encephalopathy at discharge. Patients who received lactulose experienced longer hospital stays, suggesting delayed clinical recovery. LOS: length of stay

Variable	Alcohol Abuse	No Alcohol Abuse
Patients (n)	44 (28%)	115 (72%)
Time to levocarnitine	7.8 hours	4.3 hours
Hepatic encephalopathy at discharge	0	0
Mean LOS with pharmacologic treatment	92.4 hours	-
LOS with lactulose	98.9 hours	-

## Discussion

This study highlights several important aspects of VA toxicity in a real-world clinical setting. A predominance of African American patients (75%) was observed, reflecting the demographic characteristics of the study population. Consistent with prior reports, the most common presenting feature was altered mental status, frequently associated with elevated ammonia levels, although some patients exhibited normal ammonia concentrations at presentation. These findings underscore the variability in clinical presentation and the importance of maintaining a high index of suspicion, particularly in patients with nonspecific neurological or GI symptoms [12].

Patients with multiple psychiatric or neurological disorders, often receiving concomitant medications, were found to be at increased risk for both toxicity and delayed treatment initiation. Polypharmacy and complex comorbidities may complicate diagnosis, necessitating additional investigations and prolonging the time to appropriate therapy. These observations are consistent with previous studies identifying coexisting neurologic and psychiatric conditions as significant risk factors for adverse outcomes in VA toxicity [13].

Our analysis demonstrates that early initiation of levocarnitine is associated with a more rapid reduction in ammonia levels and a shorter hospital LOS compared with initial treatment with lactulose. Patients who received lactulose prior to levocarnitine exhibited delayed biochemical improvement, while the addition of levocarnitine promptly normalized ammonia levels. These results support previous evidence indicating that lactulose is largely ineffective in VA-induced hyperammonemia and that levocarnitine remains the mainstay of treatment. Prompt recognition and removal of VA, coupled with timely levocarnitine administration, appear critical for rapid clinical recovery [14].

Alcohol use was documented in 28% of patients and was associated with delayed initiation of levocarnitine therapy and longer recovery times. Although no patient developed hepatic encephalopathy, the slower recovery among alcohol users may reflect pre-existing hepatic compromise, highlighting the need for careful monitoring and early intervention in this subgroup.

Notably, there were no fatalities in our patient cohort, emphasizing that VA toxicity is highly manageable when recognized and treated appropriately. However, limitations of this study include its retrospective design, potential selection bias, and a relatively modest sample size, which may limit generalizability. Additionally, the study could not precisely quantify delays in diagnosis, suggesting a need for future prospective investigations [15].

In conclusion, our findings reinforce the critical importance of early recognition of VA toxicity and prompt levocarnitine therapy. Lactulose does not confer a significant benefit and may delay recovery. Targeted strategies for rapid diagnosis, particularly in patients with polypharmacy or alcohol use, are essential to optimize outcomes. Further large-scale studies are warranted to validate these observations and develop standardized clinical protocols [16].

## Conclusions

VA toxicity is a significant clinical concern, particularly in patients with psychiatric or neurological comorbidities and polypharmacy. Early recognition and timely initiation of levocarnitine therapy are associated with a more rapid reduction in hyperammonemia, shorter hospital LOS, and improved clinical outcomes. Lactulose does not appear to confer additional benefit and may delay recovery when used prior to levocarnitine. Alcohol use is associated with delayed response, underscoring the need for careful monitoring. Implementation of standardized protocols emphasizing rapid diagnosis and prompt levocarnitine treatment can optimize care and reduce complications in patients with VA toxicity.
